# Proteogenomic Analysis of a Thermophilic Bacterial Consortium Adapted to Deconstruct Switchgrass

**DOI:** 10.1371/journal.pone.0068465

**Published:** 2013-07-19

**Authors:** Patrik D'haeseleer, John M. Gladden, Martin Allgaier, Patrik S. G. Chain, Susannah G. Tringe, Stephanie A. Malfatti, Joshua T. Aldrich, Carrie D. Nicora, Errol W. Robinson, Ljiljana Paša-Tolić, Philip Hugenholtz, Blake A. Simmons, Steven W. Singer

**Affiliations:** 1 Joint BioEnergy Institute, Emeryville, California, United States of America; 2 Physical and Life Sciences Directorate, Lawrence Livermore National Laboratory, Livermore, California, United States of America; 3 Biological and Materials Science Center, Sandia National Laboratories, Livermore, California, United States of America; 4 Joint Genome Institute, Walnut Creek, California, United States of America; 5 Leibniz Institute of Freshwater Ecology and Inland Fisheries, Berlin, Germany; 6 Metagenomics Applications Team, Genome Science Group, Los Alamos National Laboratory, Los Alamos, New Mexico, United States of America; 7 Biological Sciences Division, Pacific Northwest National Laboratory, Richland, Washington, United States of America; 8 Environmental Molecular Sciences Laboratory, Pacific Northwest National Laboratory, Richland, Washington, United States of America; 9 Australian Centre for Ecogenomics, School of Chemistry and Molecular Biosciences, The University of Queensland, Brisbane, Australia; 10 Earth Sciences Division, Lawrence Berkeley National Laboratory, Berkeley, California, United States of America; University of Georgia, United States of America

## Abstract

Thermophilic bacteria are a potential source of enzymes for the deconstruction of lignocellulosic biomass. However, the complement of proteins used to deconstruct biomass and the specific roles of different microbial groups in thermophilic biomass deconstruction are not well-explored. Here we report on the metagenomic and proteogenomic analyses of a compost-derived bacterial consortium adapted to switchgrass at elevated temperature with high levels of glycoside hydrolase activities. Near-complete genomes were reconstructed for the most abundant populations, which included composite genomes for populations closely related to sequenced strains of *Thermus thermophilus* and *Rhodothermus marinus*, and for novel populations that are related to thermophilic Paenibacilli and an uncultivated subdivision of the little-studied *Gemmatimonadetes* phylum. Partial genomes were also reconstructed for a number of lower abundance thermophilic *Chloroflexi* populations. Identification of genes for lignocellulose processing and metabolic reconstructions suggested *Rhodothermus*, *Paenibacillus* and *Gemmatimonadetes* as key groups for deconstructing biomass, and *Thermus* as a group that may primarily metabolize low molecular weight compounds. Mass spectrometry-based proteomic analysis of the consortium was used to identify >3000 proteins in fractionated samples from the cultures, and confirmed the importance of *Paenibacillus* and *Gemmatimonadetes* to biomass deconstruction. These studies also indicate that there are unexplored proteins with important roles in bacterial lignocellulose deconstruction.

## Introduction

Lignocellulosic biomass is an abundant feedstock for the industrial scale production of biofuels as a renewable, carbon-neutral alternative energy source, especially for high energy-density transportation fuels [1], [2]. The recalcitrance of this biomass to deconstruction into fermentable sugars is a barrier to current biofuel production efforts, and the enzymes required for biochemical deconstruction of biomass are a significant cost in the overall process [3]. Current commercial fungal enzyme cocktails may not be well suited for next-generation biomass pretreatment methods that require elevated temperatures, extreme pH, or those that generate inhibitors or residual pretreatment chemicals such as acids, bases, and/or ionic liquids [4]. Thermophilic microbes may provide a rich alternative source of glycoside hydrolases and other lignocellulolytic enzymes and pathways for biomass deconstruction that can operate efficiently under these environmental conditions [5]–[9].

Enzymes for the deconstruction of lignocellulosic biomass are most often obtained by screening of cultivated microbial isolates, primarily fungi and bacteria [10]. In natural environments, plant biomass is deconstructed by complex microbial communities that employ hydrolytic and oxidative enzymes to depolymerize polysaccharides and lignin [11]. Studying lignocellulose deconstruction by microbial communities, rather than isolates, may provide a more comprehensive view of lignocellulose deconstruction and uncover new microbial groups and deconstruction mechanisms. Natural microbial communities that deconstruct biomass are often complex and it is difficult to assign functional roles to individual microbial groups [12]. For example, microbial communities found in compost that exhibit high rates of biomass deconstruction contain a large number of taxa whose proportions are dynamically altered by changes in substrate composition and temperature [13].

Enrichment cultures established with defined substrates and at constant temperatures offer the possibility of simplifying these complex microbial communities and identifying functional roles for specific populations within the community. The feasibility of targeted discovery of glycoside hydrolases from metagenomic sequencing was demonstrated in a solid state switchgrass-adapted community [7], [14]. More recently, bacterial consortia have been adapted to switchgrass deconstruction under thermophilic conditions in liquid culture, resulting in low diversity bacterial consortia with a few dominant members and high levels of xylanase and endoglucanase activity [8]. Members of the *Firmicutes, Thermi*, and *Bacteriodetes* were the most abundant community members, with members of an uncultivated lineage of the *Gemmatimonadetes* and thermophilic *Chloroflexi* present at lower abundances. The supernatants were used to saccharify ionic liquid ([C_2_mim][OAc]) pretreated switchgrass at elevated temperatures (up to 80°C), demonstrating that this type of consortia are an excellent source of enzymes for the development of enzymatic cocktails tailored to process conditions relevant within the biorefinery context [9].

Shotgun sequencing of microbial consortia (metagenomics) is a powerful method to determine the metabolic potential of multi-species consortia without the bias inherent in microbial cultivation [15]. This method has been applied broadly to study natural and engineered microbial communities [16]. Companion proteomic measurements, referred to as community proteogenomics or metaproteomics, identify which proteins predicted by the metagenomics are produced by the microbial community [17]–[19]. Here we describe the application of proteogenomics to switchgrass-adapted communities to help define the metabolic roles of dominant uncultivated populations in the complex process of biomass deconstruction.

## Materials and Methods

### Cultivation of Switchgrass Adapted Consortia

Cultivation, DNA extraction (for metagenome sequencing), 16S rRNA community composition and enzymatic activities for the compost-derived microbial consortium adapted to switchgrass in liquid culture at 60°C (JP-9, 1% SG) were previously described [8]. Chemical characterization of the switchgrass cultivar has also been previously described [20]. Proteomic measurements were performed on a subsequent passage (JP-39, 1%SG) of the same consortium, which was maintained by 39 repeated two week passages on switchgrass at 1% solids loading that had been extracted with ethanol and hot water. The culture was split into three fractions for analysis; the solid switchgrass that settles out of the culture after 5 minutes (fraction on residual biomass), the microbial community and degraded particulate biomass suspended in the supernatant that are able to be pelleted by centrifugation at 21000 g (bacterial biomass), and the remaining supernatant that has been passed through a 0.2 μm filter (supernatant). All samples were frozen in liquid nitrogen and stored at −80°C until they were shipped on dry ice to EMSL for proteomic analysis.

### Metagenomic sequencing

Raw 454 (2,404,010 reads) and Illumina metagenomic reads (116,682,996 paired-end reads, ∼76 bp in length) were trimmed using Lucy (http://lucy.sourceforge.net/) with accuracy of 99%. Both trimmed and untrimmed reads were kept for further assembly. The 454 reads were assembled by Newbler (454 Life Sciences, CA, flags: -tr, -rip, -mi 98, -ml 80). Paired-end Illumina reads were assembled using Velvet (http://www.ebi.ac.uk/~zerbino/velvet/) at a range of Kmers (41, 43, 45, 47, 49, 51, 53, 55, 57) for both trimmed and untrimmed reads. Default settings for all Velvet assemblies were used (flags: -exp_cov auto). The best contigs from Velvet (untrimmed kmer 43) were selected from 18 total contig sets based on the number of contigs, max contig size and total size. Contigs larger than 2000 bp were then shredded into 1800 bp with 900 bp overlap reads. Based on the same metrics, the untrimmed 454 reads gave better assembly. In an attempt to generate larger contigs, the raw 454 data plus shredded Illumina contigs were assembled by Newbler again for the final assembly.

Assembled metagenomic sequences were uploaded into the DOE Joint Genome Institute's Integrated Microbial Genomes with Microbiome Samples – Expert Review (IMG/M-ER) system [21] and annotated according to the Standard Operating Procedure for the Annotations of Genomes and Metagenomes submitted to the Integrated Microbial Genomes Expert Review (IMG-ER) System [22]–[24].

The protein sequences were also screened for carbohydrate-active enzymes using dbCAN [25] and using blastp against a local copy of the CAZy [26] and FOLy [27] databases (E<1e^−20^), keeping track of both the best blastp hit overall, and the best hit against any protein in CAZy or FOLy with an experimentally validated EC number. To achieve better coverage of laccase-like enzymes, the annotated sequences were additionally screened against a small set of known bacterial laccases [28], and a larger set of 1240 bacterial laccase-like enzymes [29].

### Phylogenetic binning

Contigs were screened for the presence of phylogenetic markers, based on the phylogenetic marker COGs identified by IMG. Phylogenetic markers were assigned to the genus of their closest blastp hit (E<1e^−20^) against NR, and contigs were assigned to the genus of the majority of their phylogenetic markers. All contigs ≥1000 bp were scored against these seed phylogenetic bins by ClaMS [30], using De Bruijn Chains with a length of 3. In the first round, contigs were assigned to their best (i.e., lowest) scoring bin, provided the score was <0.01, and the second best score was no more than 120% of the top score, in order to avoid binning contigs that fell between two bins. Some bins actually decreased in size due to this latter requirement, indicating that the oligonucleotide profile of the seed bins was too similar to other bins. These seed bins were merged or deleted, and the contigs were rescored against the new set of seed bins. The resulting enlarged bins were then used to classify all contigs ≥1000 bp to their best scoring phylogenetic bin.

Each phylogenetic bin was then examined individually to identify tight clusters of contigs centered on a specific read coverage, corresponding to genome reconstructions for specific populations present within the microbial community. Average amino acid identity (AAI, [31]) for these clusters against sequenced reference genomes was calculated based on the best one-way BLAST hits with at least 30% sequence identity over 70% of the length of the gene. Completeness of the genome coverage within these clusters was estimated based on what fraction of a set of 54 single-copy or almost single-copy phylogenetic COGs (see File S3) was covered within the cluster, weighting the 30 ribosomal COGs among these as 1/30^th^ of a nonribosomal COG, because the former typically co-occur in large gene clusters and therefore should not be counted as independent measures for the completeness of the genome reconstruction.

### Metabolic modeling

Pathway genome databases (PGDBs) were constructed for each phylogenetic bin using SRI International's Pathway Tools platform v14.0 [32], treating each bin as a draft genome in many contigs. To minimize gaps or missing pathways the entire bin was included rather than attempting to separate out strains with different read coverage. The pathway genome databases have undergone minimal manual curation and may contain some errors, similar to a Tier 3 BioCyc PGDB [33]. Pathway enrichment analysis for proteins significantly differentially represented in the planktonic versus fiber attached cellular protein fraction was performed using the Enrichment Analysis tool built into Pathway Tools, using the Fisher exact test across all pathways and pathway classes, with a Benjamini-Hochberg correction for multiple hypothesis testing [34], at a False Discovery Rate of 10%.

### Preparation of Reference Database for Protein Identification by Mass Spectrometry

A non-redundant proteomics reference database was prepared from the metagenomic sequence data by sorting all 63,824 annotated protein sequences based on the read coverage of the contigs (contigs with higher coverage are less likely to contain sequencing errors), protein length (longer sequences are less likely to be truncated by an incorrect gene call or frame shift sequencing error), and penalty terms for genes within 200bp of either end of the contig:
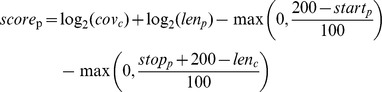



The sorted protein sequences were subsequently dereplicated using UCLUST at 95% sequence identity, such that among clusters of highly similar protein sequences, only the highest scoring, and thus most reliable sequence, was retained.

### Sample Preparation for Proteomic Analysis

Water was added to the bacterial pellet with 1 mM PMSF, vortexed and added to a barocycle pulse tube (Pressure Biosciences Inc., South Easton, MA). Water with 1 mM PMSF was also added to the switchgrass in an ice cold mortar and pestle and ground in liquid nitrogen for 2 minutes before being added to a barocycle pulse tube and both samples were barocycled for 10 cycles (20 seconds at 35,000 psi back down to ambient pressure for 10 seconds) for cell lysis. These samples were removed and along with the supernatant sample; a chloroform methanol extraction was performed in two separate sets for each fraction by adding a 5∶1 ratio over sample volume of ice cold (−20°C) chloroform: methanol mix (prepared 2∶1 (v/v)). Samples remained on ice for 5 minutes followed by rigorous vortexing and centrifuging at 12,000 g for 10 minutes. The upper and lower phases were collected and stored, leaving the protein interphase. Ice cold methanol was added to the protein, vortexed and centrifuged to collect the pellet (and dried to remove the remaining methanol). To one set of the collected the protein phases, a 2,2,2-Trifluoroethanol (TFE) based protocol [35] was performed and to the other set, a commonly used 8 M urea protocol [36] was performed for sample preparation. For the TFE digestion, protein pellets were reconstituted in 250 µl of 100 mM ammonium bicarbonate (NH_4_HCO_3_), pH 8.0 and assayed with Bicinchoninic acid (BCA) (Thermo Scientific, Rockford, IL) to determine the protein concentration. TFE (Sigma, St. Louis, MO) was added to the sample for a final concentration of 50% TFE. The sample was sonicated in an ice-water bath for 1 minute and incubated at 60°C for 2 hours with gentle shaking. The sample was then incubated with 2 mM dithiothreitol (DTT) (Sigma, St. Louis, MO) at 37°C for 1 hour with gentle shaking. Samples were diluted 5-fold with 50 mM (NH_4_HCO_3_) for preparation for digestion. CaCl_2_ (1 mM) and sequencing-grade modified porcine trypsin (Promega, Madison, WI) were added to all protein samples at a 1∶50 (w/w) trypsin: protein ratio for 3 h at 37°C. The sample was concentrated in a Speed Vac (ThermoSavant, Holbrook, NY) to a volume of ∼30 µl and was then centrifuged at 14,000 rpm. These samples were used for the 1D-LC-MS analysis described below. The remaining protein pellets were resuspended in 8 M urea and 10 mM DTT sonicated and incubated at 60°C for 30 min with constant shaking. Samples were then diluted 10-fold for preparation for digestion with 100 mM NH_4_HCO_3_; 1 mM CaCl_2_ and sequencing-grade modified porcine trypsin (Promega, Madison, WI) was added to all protein samples at a 1∶50 (w/w) trypsin: protein ratio for 3 h at 37°C. The samples (along with an equal portion of the TFE digested sample) were cleaned using Discovery C-18 50 mg/1 mL solid phase extraction tubes (Supelco, St.Louis, MO) using the following protocol: 3 mL of methanol was added for conditioning followed by 2 mL of 0.1% TFA in H_2_O. The samples were then loaded onto each column followed by 4 mL of 95:5: H_2_O: acetonitrile (ACN), 0.1% TFA. Samples were eluted with 1 mL 80∶20 ACN: H_2_O, 0.1% TFA. The samples were concentrated down to ∼100 µL using a Speed Vac and a final BCA assay was performed to determine the peptide concentration. These samples were then placed in vials for 2D-LC-MS mass spectrometric analysis described below.

### 1D-LC-MS Analysis

Samples of digested proteins from the supernatant, cell pellet, and extracted from culture solids, e.g. switchgrass, were analyzed by 1D RPLC MS using a custom constant pressure system with a 75 µm i.d. by 70 cm length column packed in-house with 3 µm Jupiter C-18 (Phenomenex, Torrance, CA) reverse phase particles. A 100 minute exponential gradient was used with water with 0.1% formic acid as solvent A and acetonitrile with 0.1% formic acid as solvent B. Mass spectrometry data was acquired using an LTQ Orbitrap (Thermo Scientific, San Jose, CA) outfitted with a custom electrospray ionization (ESI) interface. Electrospray emitters were custom made using 150 µm o.d. ×20 µm i.d. chemically etched fused silica [37]. The heated capillary temperature and spray voltage were 200°C and 2.4 kV, respectively. Data was acquired for 100 min, beginning 65 min after sample injection and 15 min into gradient. Orbitrap spectra were collected from 400–2000 *m*/*z* at a resolution of 100 k followed by data dependent ion trap CID MS/MS of the six most abundant ions. A dynamic exclusion time of 180 sec was used to discriminate against previously analyzed ions.

### 2D-LC-MS Analysis

The 2D-LC system was custom built using two Agilent 1200 nanoflow pumps and one 1200 capillary pump (Agilent Technologies, Santa Clara, CA), various Valco valves (Valco Instruments Co., Houston, TX), and a PAL autosampler (Leap Technologies, Carrboro, NC). Full automation was made possible by custom software that allows for parallel event coordination providing near 100% MS duty cycle through use of two trapping and analytical columns. All columns were manufactured in-house by slurry packing media into fused silica (Polymicro Technologies Inc., Phoenix, AZ) using a 1 cm sol-gel frit for media retention [38]. First dimension SCX column; 5 µm PolySULFOETHYL A (PolyLC Inc., Columbia, MD), 15 cm×360 µm o.d. ×150 µm i.d. Trapping columns; 5 µm Jupiter C-18 (Phenomenex, Torrence, CA), 4 cm×360 µm o.d. ×150 µm i.d. Second dimension reversed-phase columns; 3 µm Jupiter C-18 (Phenomenex, Torrence, CA), 35 cm×360 µm o.d. ×75 µm i.d. Mobile phases consisted of 0.1 mM NaH_2_PO_4_ (A) and 0.3 M NaH_2_PO_4_ (B) for the first dimension and 0.1% formic acid in water (A) and 0.1% formic acid in ACN (B) for the second dimension.

MS analysis was performed using a LTQ Orbitrap Velos ETD mass spectrometer (Thermo Scientific, San Jose, CA) outfitted with the custom ESI interface described above. The heated capillary temperature and spray voltage were 275°C and 2.2 kV, respectively. Data was acquired for 100 min, beginning 65 min after sample injection and 15 min into gradient. Orbitrap spectra were collected from 400–2000 *m/z* at a resolution of 100 k followed by data dependent ion trap CID MS/MS of the ten most abundant ions. A dynamic exclusion time of 180 sec was used to discriminate against previously analyzed ions.

### Accurate Mass and Time (AMT) tag analysis

The accurate mass and time (AMT) tag approach [39]–[41] was used to generate quantitative peptide peak intensity data. This method is an LC-MS approach which works by directly matching LC-MS features to a previously generated feature database using accurate mass and LC elution time information. The AMT database is generated by 2-D analysis of samples using LC-MS/MS to identify peptides and associated accurate mass and LC elution time for inclusion. Peptides sequences were identified using the SEQUEST v.27 (rev. 12) search engine and re-scored using MS-GF (v6432) scoring. The feature database was populated using identifications having a spectral probability of 10^−9^ or lower, partial/full trypticity and a Peptide Prophet score ≥0.5. Features from the 1-D analysis were matched to this database and filtered using a uniqueness probability of 0.51 to ensure specificity of the match.

For derivation of protein abundances from AMT peptide measurements, we used the QRollup method available in DAnTE [42]. Briefly, peptides are selected on the basis of a user selected abundance cutoff value (in this case, the top 33%) as well as unique association to a single protein, and protein abundance is calculated as the average of these selected peptides.

### Normalization and statistical analysis of proteome data

We normalized protein abundances by dividing by a size factor for each replicate, as proposed by Anders and Huber [43], such that the median fold change for the proteins that occur in all three replicates of a protein fraction approximates one:
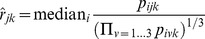
where 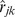 is the size factor for replicate *j* of protein fraction *k,* and 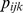 is the protein abundance for gene *i* in that replicate.

After normalizing each replicate within the protein fractions to each other, we calculate the average protein abundance within each fraction, and normalize the fractions against each other in a similar way, by dividing by a fraction-specific size factor based on the average abundance for the 461 proteins that occur in every replicate in every protein fraction:
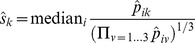
where 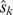 is the size factor for protein fraction *k,* and 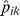 is the average protein abundance for gene *i* in that fraction, after normalizing by dividing each replicate by the size factor 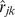 described above, i.e. 
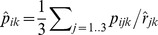
.

The resulting normalized protein abundances were used to search for proteins that were significantly overexpressed by at least 2-fold in the supernatant fraction compared to the cellular fractions. Because of the nature of proteomics data, there are a large number of missing values in the data, meaning that it was not possible to accurately quantitate any peptide peaks for some proteins in some replicates. “Missingness” in proteomics data is typically associated with below-threshold peak intensities, so averaging only across non-missing values will overestimate protein abundance [44], [45]. Conversely, setting missing values equal to zero or to the detection threshold will tend to underestimate the variance of the data. Here we take a hybrid approach to dealing with missing values, using the detection threshold to estimate protein abundance averages, and a pooled variance estimate extrapolated for that average.

To calculate average protein abundance within a protein fraction, missing values were conservatively set to 10,000, which is slightly below the detection threshold (the lowest measured protein abundance after normalization is equal to 13,111). For proteins without any missing values, we can use the variance between the replicates within each fraction to perform a T-test for significant over- or under-representation between fractions. For proteins with only a single non-missing value in a protein fraction, we use a pooled variance estimate, by fitting a power law to the variance as function of the mean abundance, across all proteins without missing values. For proteins with only a single missing value, we take the average of the per-protein variance and the pooled variance.

### Data Archiving

Raw sequencing reads are available in the NCBI Short Read archive under accession SRX122233, SRX040418 and SRX11792. Assembled metagenome scaffolds, gene calls and functional annotations are available through the DOE Joint Genome Institute's Integrated Microbial Genomes and Metagenomes (IMG/M) system [21] under Taxon Object ID 2061766001(“Thermophilic enrichment culture SG0.5JP960 (454-Illumina assembly) – version 2 (454-Illumina assembly v2)”). MS proteomics data is available at http://omics.pnl.gov/view/publication_1072.html. Metabolic pathway/genome databases for the reconstructed genomes corresponding to the phylogenetic bins are available at http://downloads.jbei.org/data/microbial_communities/proteogenomics/.

## Results

### Metagenomic Sequencing and Assembly

Metagenomic sequencing was performed on a switchgrass-adapted thermophilic culture whose community composition and glycoside hydrolase activities were previously described (JP-1% SG) [8]. Combined assembly of ∼10 Gbp of 454 and Illumina reads using Velvet and Newbler resulted in a total of 49,664,259 bp of assembled sequence in 20,206 scaffolds of length 100 to 389,333 bp, including 9,413 of length 1 Kbp or larger, with an N50 of 5936 bp and average GC content of 64.5%. Annotation by the IMG/M pipeline identified a total of 64,668 genes, including 844 RNA genes and 63,824 protein coding genes, of which 68% were assigned a putative function. See File S1 for the full metagenomic sample information.

### Phylogenetic Binning

**Figure 1:**
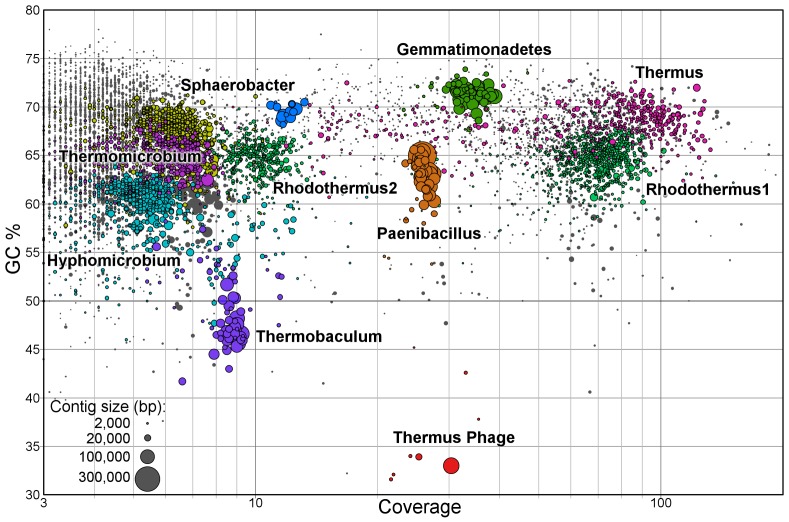
Each contig was plotted against its average read coverage per base (X axis), and its GC% content (Y axis). The surface area of each circle is proportional to the length of the contigs in bp, giving an intuitive visualization of how much metagenomic sequence is covered by each cluster. Phylogenetic bins are represented by different colors, while grey circles represent (typically smaller) contigs that were not assigned to a bin.

Phylogenetic binning was performed on the metagenomic assembly and 10 bins were classified from 5198 contigs that cover 36 MB (73%) of the total assembled sequence (Figure 1, and File S1). Most of the phylogenetic bins clustered around a narrow range of read coverages, each likely corresponding to a discrete population within the microbial community. These bins also contained sets of smaller contigs at much lower read coverage, which may correspond to lower abundance strains or sequences that were not as well-assembled. Phylogenetic assignments of the bins were consistent with reconstructed 16S rRNA sequences obtained by the EMIRGE method [8]. Average read coverage of the metagenomic clusters provides a more direct measure of abundance of the populations in the microbial community, whereas 16S rRNA gene abundance may be incorrectly estimated by more than an order of magnitude due to large differences in 16S rRNA gene copy number for the lineages in this community (see File S2) [46]. Genes from the two most abundant phylogenetic bins (70–75x average coverage) were closely related to sequenced strains of *Thermus thermophilus* and *Rhodothermus marinus* [47], [48], with average amino acid identity (AAI, [31]) of 91% and 96% respectively (Table 1). The relatively poor metagenome assembly for the *Thermus* bin compared to populations with lower abundance could be partially explained by the observation of polyploidy [49] and hyperplasticity [50] in *Thermus* species, which may allow for some nucleotide diversity.

**Table 1:** Phylogenetic bins and statistics.

Bin	covg	GC%	Size bp	contigs	N50	Best reference genome	AAI	Completeness^c^
***Thermus***	74.7	68%	2,126,834	457	5,482	*T. thermophilus* HB27	91%	72%
***Rhodothermus*** ** 1**	71.2	65%	2,899,900	732	4,581	*R. marinus* DSM 4252	96%	96%
***Gemmatimonadetes***	34.5	71%	3,785,002	87	64,448	*G. aurantiaca* T-27	48%	100%
***Thermus*** ** phage**	29.2	34%	168,710	8	123,990	*T. thermophilus* phage YS40	84%	99%^a^
***Paenibacillus***	26.1	63%	2,985,204	48	89,045	*Paenibacillus sp.* JDR-2	56%	96%
***Rhodothermus*** ** 2**	10.1	65%	1,689,164	393	5,280	*R. marinus* DSM 4252	95%	60%
***Thermobaculum***	8.7	48%	2,237,622	71	59,820	*T. terrenum* ATCC BAA-798	98%	88%
***Sphaerobacter*** **^ b^**	6.3	67%	5,207,803	662	10,556	*S. thermophilus* DSM 20745	85%	80%
***Thermomicrobium***	6.2	65%	2,635,886	397	8,832	*T. roseum* DSM 5159	76%	87%
***Hyphomicrobium***	5.7	60%	4,323,820	546	11,083	*H. denitrificans* ATCC 51888	56%	90%

a : Based on blastn coverage of reference genome, rather than phylogenetic markers. ^b^: Including additional cluster at 12.0 read coverage that likely corresponds to a Sphaerobacter megaplasmid. ^c^: See also File S3.

Two other abundant bins clustered with Gram-positive thermophilic *Bacilli* (*Paenibacillus* and *Thermobacillus*) and an uncultivated lineage in the *Gemmatimonadetes* phylum [51]. The *Paenibacillus* bin only covers 2.99 Mb of sequence, less than half the length of the closest reference genome, *Paenibacillus* sp. JDR-2 (7.18 Mb). However, the reconstructed genome is estimated to be 96% complete, based on the presence of universal phylogenetic markers (see File S3), and it contains 88% of the COGs that are present in single copy in six publicly available complete *Paenibacillus* genomes available in IMG, strongly suggesting that this cluster corresponds to an unusually small yet nearly complete *Paenibacillus* genome. *Gemmatimonadetes* is wide-spread, particularly in terrestrial habitats, making up on average 2% of soil bacterial communities [52], [53], and has a phylogenetic breadth that is greater than the *Actinobacteria* (19% *vs.* 18% 16S rDNA sequence divergence [51]). The strain present in the switchgrass enrichment community belongs to subdivision five of the *Gemmatimonadetes* phylum [8], and is distantly related (88% 16S rDNA sequence identity, AAI 48%) to the only sequenced representative of this phylum, *Gemmatimonas aurantiaca* T-27 [51].

At lower coverage, a clearly distinguished second bin related to *Rhodothermus marinus* was also observed at 10x sequence coverage, with 95% average amino acid identity to homologous genes in the dominant *Rhodothermus* bin. Lower abundance bins were assigned to members of the *Chloroflexi* (*Sphaerobacter, Thermobaculum, Thermomicrobium*) and *α*-proteobacteria (*Hyphomicrobium*). The bin assigned to *Thermobaculum* had 98% average amino acid identity to a sequenced strain of *Thermobaculum terrenum* [54]. *Sphaerobacter thermophilus*, the closest relative to the *Sphaerobacter* bins, has two replicons (2.7 Mbp and 1.2 Mbp) [55], the larger of which is closely related to the 4.6 Mbp cluster at ∼6x coverage. Another small cluster with an average GC content of ∼70% and 12x coverage (blue cluster in Figure 1) consists of contigs that do not contain phylogenetic markers and were originally assigned to *Sphaerobacter* and *Conexibacter* based on phylogenetic binning, but may instead correspond to the small chromosome of *S. thermophilus*. We hypothesize that these sequences may represent a megaplasmid in the *Sphaerobacter* population present in this community, which might explain the absence of conserved marker genes and 2x difference in coverage compared to the main cluster of *Sphaerobacter* contigs.

### Genes Involved in Biomass Deconstruction

Analysis of genome annotations, including cataloguing of glycoside hydrolase (GH) genes (Table 2, and File S4), and metabolic pathway reconstructions, provided putative roles for the abundant community members. The *Thermus thermophilus*-like phylogenetic bin contained a small number of lignocellulolytic enzymes, including three putative beta-glycosidases (GH1, GH42) and two putative laccases. The bins related to *Rhodothermus marinus* contained one of the highest number of glycoside hydrolase genes in the reconstructed genomes from the switchgrass adapted community (63 in the *Rhodothermus*1, 38 in *Rhodothermus*2), including three GH5 endoglucanases, 13 β-glucosidases, and at least 15 hemicellulases. This glycoside hydrolase profile is very similar to what is contained in the genome of *R. marinus* DSM 4254 [48]. The *Rhodothermus*-like genomes also have one of the highest complements of carbohydrate degradation pathways (11 pathways, involving 26 genes from *Rhodothermus1*), including pectin (homogalacturonin) and pentose degradation. The *Gemmatimonadetes* bin has genes annotated for cellulose hydrolysis (GH1, GH3, GH9) and multiple laccases, consistent with a possible role in lignin degradation. 29% of the carbohydrate and lignin active enzymes in this bin have no homolog (>30% amino acid identity) to *G. aurantiaca* T-27. The *Paenibacillus* bin contains one of the highest number of GHs (57 in the top cluster), including three endoglucanases, two cellobiohydrolases, and a large complement of carbohydrate degradation pathways (9 pathways, involving 26 genes), indicating that it is a highly versatile cellulose and hemicellulose degrader. 22% of the carbohydrate and lignin active enzymes in this bin have no homolog to the closest sequenced reference organism, *Paenibacillus sp. JDR-2*.

**Table 2:** Carbohydrate active enzymes and ligninases in metagenome (see also File S4).

Bin	GH	CBM	selected GH's	selected enzymes
**Thermus**	18	2	1 GH1, 1 GH9, 2 GH42	1 β-Glucosidase, 2 β-Galactosidase, 2 Laccase,
**Rhodothermus1**	63	18	2 GH2, 6 GH3, 2 GH5, 1 GH16, 2 GH35, 1 GH43, 2 GH67	2 Endoglucanase, 8 β-Glucosidase, 1 Endoxylanase, 3 β-Xylosidase, 4 α-L-Arabinofuranosidase, 2 β-Galactosidase, 2 Laccase
**Gemmatimonadetes**	37	13	1 GH1, 1 GH2, 3 GH3, 2 GH9, 1 GH74	1 Endoglucanase, 2 β-Glucosidase, 5 Laccase
**Paenibacillus**	57	23	1 GH1, 4 GH2, 4 GH3, 1 GH5, 1 GH8, 1 GH9, 1 GH11, 1 GH39, 1 GH42, 3 GH43, 1 GH67	3 Endoglucanase, 2 CBH2, 4 β-Glucosidase, 6 Endoxylanase, 5 β-Xylosidase, 5 α-L-Arabinofuranosidase, 2 β-Galactosidase, 1 Feruloyl esterase, 3 Laccase
**Rhodothermus2**	38	18	1 GH2, 5 GH3, 1 GH5, 1 GH12, 1 GH16, 1 GH67	1 Endoglucanase, 5 β-Glucosidase, 1 β-Xylosidase, 1 α-L-Arabinofuranosidase, 2 Laccase
**Thermobaculum**	44	6	1 GH2, 3 GH3, 2 GH4, 1 GH6	2 Endoglucanase, 2 β-Glucosidase, 1 Endoxylanase, 1 β-Xylosidase, 1 α-L-Arabinofuranosidase, 2 Laccase
**Sphaerobacter**	100	17	3 GH1, 3 GH2, 1 GH3, 2 GH4, 5 GH5, 3 GH29, 9 GH39, 2 GH42, 4 GH43, 23 GH109	2 Endoglucanase, 4 β-Glucosidase, 1 α-L-Arabinofuranosidase, 1 β-Galactosidase, 1 Feruloyl esterase, 9 Laccase
**Thermomicrobium**	26	8	2 GH1, 1 GH4, 1 GH5	3 Endoglucanase, 2 β-Glucosidase, 1 Endoxylanase, 3 Laccase
**Hyphomicrobium**	31	5	1 GH9, 1 GH11, 1 GH39	1 Endoglucanase, 1 Laccase, 11 glutathione S-transerases (potential beta-aryl etherases)

Among the lower abundance bins, GHs for cellulose hydrolysis were found in the *Chloroflexi*, especially the *Sphaerobacter* bins, which contained the highest number of GHs (100, including 5 GH5) and the highest complement of carbohydrate degradation pathways (12 pathways, involving 29 genes). The *Sphaerobacter* bins also contained the largest number of putative laccases (9) and a large number of aromatic degradation pathways (13 pathways, involving 14 genes), including a 4-hydroxyphenylacetate degradation pathway which has been implicated in metabolizing lignin degradation products [56]. The *Hyphomicrobium* bin contained the highest number of aromatic degradation pathways (20 pathways involving 24 genes, compared to only 5 carbohydrate degradation pathways involving 10 genes) including ortho-cleavage of protocatechuate, suggesting that it may specialize in lignin catabolism. It also contains 11 glutathione S-transferases, a family of xenobiotic degradation enzymes that is known to include proteins involved in cleavage of beta-aryl linked lignin dimers [57]. Across the entire metagenome, close to 10% of all genes are annotated to be involved in transport (from 8% for *Gemmatimonadetes* to 13% for *Hyphomicrobium*), and one fifth of these transporters are predicted to be involved in carbohydrate transport. The *Gemmatimonadetes* bin has the smallest fraction of annotated carbohydrate transporters (0.5%), while the *Paenibacillus* bin has by far the highest number of carbohydrate transporters (3.4% of all genes and 30% of all its annotated transporters). The *Rhodothermus1* and *Thermobaculum* bins also contain a relatively high fraction of sugar isomerases: 22/3336 (0.7%), and 23/2269 (1%), respectively, compared to 0.2–0.5% for the other phylogenetic bins.

### Proteomics

The proteome of the switchgrass-adapted consortium was analyzed by fractionating the culture into three components: the soluble fraction (supernatant), the suspended fraction (bacterial biomass) and the attached fraction (fraction on residual biomass). These fractions were digested with trypsin and analyzed by 2D-LC-MS to obtain a comprehensive survey of proteins present in the fractions and 1D-LC-MS with accurate mass tags for quantitative protein comparisons. The peptides were compared to a database of predicted peptides from the metagenomic data. A total of 4148 proteins were identified from all three fractions, representing 6.5% of the total predicted proteins from the metagenome (File S5). Of these proteins, 338 were unique to the supernatant fraction, 1108 to the suspended fractions and 409 to the attached fraction.

**Figure 2:**
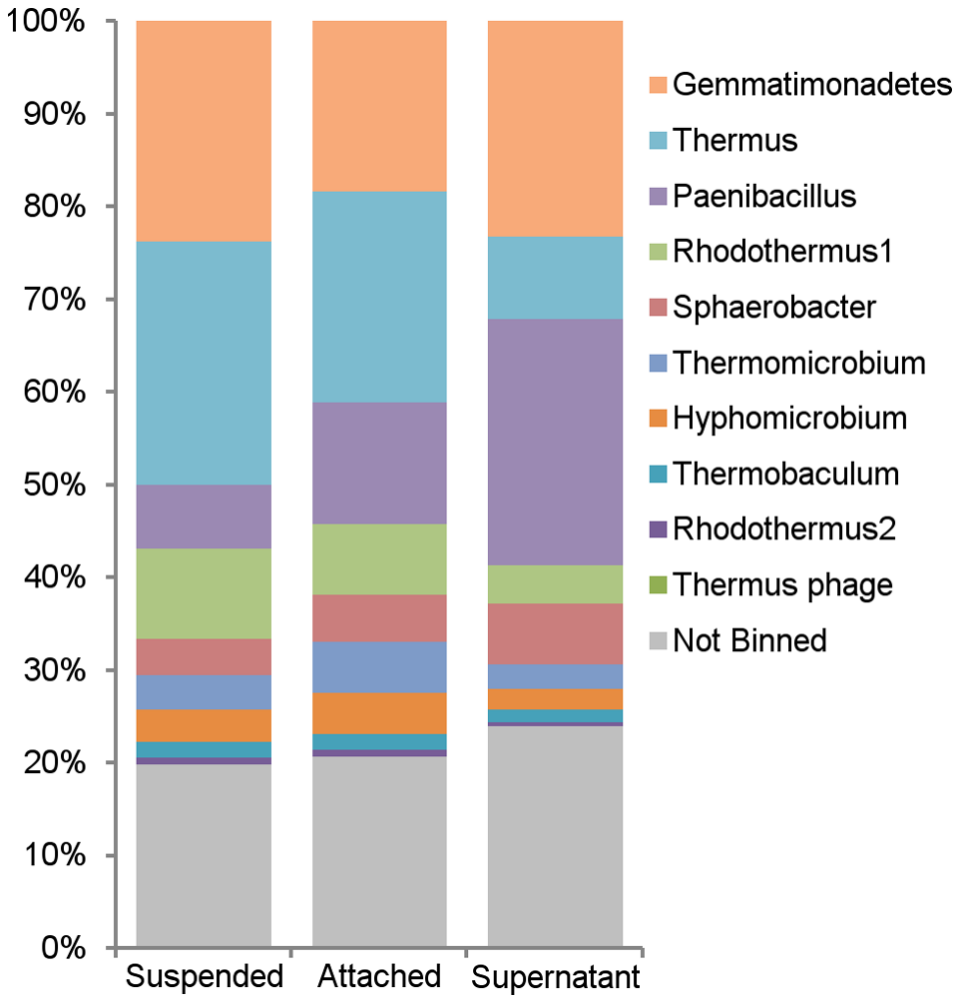
Normalized protein abundance within each of the three proteome fractions, by phylogenetic bins.

The proteins were assigned to specific bins based on comparison to the metagenomic data. In all three fractions, proteins assigned to the *Gemmatimonadetes, Paenibacillus* and *Thermus* bins were >50% of the total estimated protein abundance (Figure 2). While among the most abundant bins in the metagenome, proteins assigned to *Rhodothermus* seem to be much less prevalent in the proteome, accounting for only 4–10% of the proteome fractions. Statistical analysis of the proteome (see Materials and Methods) revealed that proteins in the *Thermus* bin were dominant in the suspended and the fiber-attached fractions, but underrepresented in the supernatant fraction. In contrast, proteins from the *Paenibacillus* bin were especially overrepresented in the supernatant fraction, constituting >26% of the proteome. Proteins from the *Sphaerobacter* bin, which was among the minor bins in the metagenome, were overrepresented in the supernatant.

Analysis of specific proteins in the proteome focused on the supernatant fraction, as proteins associated with biomass deconstruction are often observed in the extracellular fraction of bacteria and the supernatants from these enrichments have been used successfully to saccharify pretreated switchgrass [8]. Among the proteins which were most abundant and overrepresented in the supernatant (Table 3, and File S5), three of the top ten belong to the CUT1 family of carbohydrate ABC transporter substrate-binding protein, all of which are related to proteins expressed by *Paenibacillus* isolates. In Gram positive bacteria, these extracellular solute binding proteins are typically bound to the external surface of the cell membrane via an N-terminal lipid anchor [58], however these proteomics data suggest they may also be released in the medium (as was demonstrated for the OppA peptide-binding protein in *B. subtilis* [58]), where they may play a role interacting with oligosaccharides. Only one of the ten most abundant overrepresented supernatant proteins, an esterase with a carbohydrate binding module from family 9, assigned to the *Paenibacillus* population, was implicated in biomass hydrolysis. Fifty-six proteins (1.3% of all proteins detected) annotated as glycoside hydrolases involved in biomass deconstruction were identified across all three fractions (Table 4, and File S5). The majority of these proteins were involved in deconstruction of hemicellulose (GH10, 51, 74, 2) or starch (GH13, 31). There were relatively few detected proteins that are involved in cellulose hydrolysis. Of these, two endoglucanases, a GH5 and a GH9, were assigned to *Paenibacillus*, one GH9 family putative endoglucanase was assigned to *Gemmatimonadetes*. Additionally, there were 6 GH3 and 4 GH1 proteins that may be β-glucosidases. Surprisingly, proteins annotated as xylose, arabinose, and sugar or sugar phosphate isomerases, normally considered to be cytoplasmic enzymes, were overrepresented by an average of 4–10 fold in the supernatant, whereas other classes of isomerases show no such fraction specificity (File S5).

**Table 3:** Top 10 most abundant overrepresented proteins in the supernatant (see also File S5).

Locus tag	Protein Description	Bin	Supernatant	Fold
sg4i_00021160	carbohydrate ABC transporter substrate-binding protein, CUT1 family (TC 3.A.1.1.-)	Paenibacillus	2.48E+09	84
sg4i_00251940	hypothetical protein	Gemmatimonadetes	1.49E+09	27
sg4i_00325360	S-layer homology domain.	Paenibacillus	5.34E+08	6.8
sg4i_00645210	carbohydrate ABC transporter substrate-binding protein, CUT1 family (TC 3.A.1.1.-)	Paenibacillus	3.92E+08	42
sg4i_00646120	Enterochelin esterase and related enzymes	Paenibacillus	3.2E+08	31
sg4i_00319610	carbohydrate ABC transporter substrate-binding protein, CUT1 family (TC 3.A.1.1.-)	Paenibacillus^a^	2.78E+08	19
sg4i_00608910	hypothetical protein	Rhodothermus^a^	2.65E+08	14
sg4i_00027040	Xaa-Pro aminopeptidase	Paenibacillus	2.57E+08	9
sg4i_00336790	Predicted transcriptional regulator	Hyphomicrobium	2.49E+08	49
sg4i_00622770	hypothetical protein	Thermus^a^	2.42E+08	163

a : Not binned; assigned based on best blastp hit.

**Table 4:** Overrepresented lignocellulolytic enzymes in the supernatant (see also File S5).

Locus tag	Protein Description	Family	EC	Bin	Supernatant	Fold
sg4i_00646120	Enterochelin esterase and related enzymes	CBM9	3.1.1.73	Paenibacillus	3.20E+08	31
sg4i_00255110	Putative multi-copper oxidases	Laccase	1.10.3.2	Gemmatimonadetes	1.27E+08	150
sg4i_00193360	BNR/Asp-box repeat.	GH74		Sphaerobacter	6.59E+07	6591
sg4i_00004420	Alpha-L-arabino-furanosidase	GH51	3.2.1.55	Paenibacillus	6.27E+07	8.1
sg4i_00024910	Beta-1,4-xylanase	GH10, CBM4/9	3.2.1.8	Paenibacillus	2.85E+07	4.3
sg4i_00608930	Beta-1,4-xylanase	GH10, CBM4/9	3.2.1.8	Hyphomicrobium^a^	1.59E+07	2.7
sg4i_00588670	Carbohydrate binding domain.	CBM16		Paenibacillus	1.12E+07	3.1
sg4i_00324270	Endoglucanase	GH5	3.2.1.4	Paenibacillus	9.65E+06	29
sg4i_00432730	maltooligosyl trehalose hydrolase (EC 3.2.1.141)	GH13, CBM48	3.2.1.141	Sphaerobacter	5.39E+06	43
sg4i_00141500	Alpha-L-fucosidase	GH29	3.2.1.51	Sphaerobacter	4.72E+06	472
sg4i_00592760	Alpha-glucosidases, family 31 of glycosyl hydrolases	GH31	3.2.1.-	Paenibacillus	2.93E+06	15
sg4i_00102640	Alpha-glucosidases, family 31 of glycosyl hydrolases	GH31	3.2.1.-	Rhodothermus	2.56E+06	3.4

a : Not binned; assigned based on best blastp hit.

Few proteins known to be involved in lignin deconstruction were detected in the proteome, although this could be due in part to our relative lack of knowledge of lignin deconstruction in bacteria [59]. However, a putative laccase assigned to the *Gemmatimonadetes* population was one of the most abundant and overrepresented proteins in the supernatant sub-proteome. In addition, a number of enzymes involved in reactions with reactive oxygen species were detected in the supernatant, suggesting oxidative degradation of the lignocellulosic biomass. This includes a superoxide dismutase from the *Hyphomicrobium*-like population and a Mn-containing catalase from the *Paenibacillus* which were highly expressed in the supernatant but nearly absent in the other proteome fractions.

## Discussion

Applying proteogenomic methods to a thermophilic bacterial consortium adapted to grow on switchgrass has revealed functional roles for community members and identified proteins that may be involved in switchgrass deconstruction. Analysis of metagenomic sequencing data identified the most abundant populations, as measured by read depth of assembled contigs, as closely related to sequenced strains of *Thermus thermophilus* and *Rhodothermus marinus*. Interestingly, the fraction of the proteome assigned to *Rhodothermus* (8%) was lower than proteins assigned to *Paenibacillus* (16%) and *Gemmatimonadetes* (22%), which were at lower relative abundance in the metagenome. This discrepancy may have arisen because the proteome measurements were performed on a later passage of the switchgrass adapted community, and the relative ratios of the abundant populations has been shown to fluctuate in switchgrass-adapted enrichments [60], but could also reflect differences in relative activity.

The *Thermus* and *Rhodothermus* populations were closely related to sequenced organisms; however, the sequences binned as *Paenibacillus* and *Gemmatimonadetes* were not closely related to sequenced isolates and the reconstructed genomes represent novel composite genomes, with average amino acid identity of 56% and 48% respectively, including a large number of novel carbohydrate or lignin active enzymes. This novelty, combined with the fact that three of the closest reference genomes (*R. marinus*, *T. terrenum*, and *S. thermophilus*) were only recently sequenced as part of the Genomic Encyclopaedia of Bacteria and Archaea project [61], also highlights the need for further sequencing of reference genomes in poorly represented phyla to improve metagenomic analyses. Phylogenetic analysis of the 16S rRNA gene recovered from the *Gemmatimonadetes* population in the adapted consortium indicates that it is affiliated with subdivision 5 of the phylum (Gemm-5) [8], the first genomic representation for this subdivision. The composite genome reconstructed for the *Gemmatimonadetes* population showed that it is substantially divergent from the sole reference isolate and genome for this phylum, *Gemmatimonas aurantiaca* [51], which belongs to a separate subdivision (subdivision 1), comparable with the divergence of alpha- and gamma-proteobacteria. The majority of *Gemmatimonadetes* 16S rRNA gene sequences have been recovered from soil, with the highest proportion found in more arid soils [52]. *Gemmatimonadetes* 16S rRNA gene sequences have been recovered from compost samples; however these sequences were at low abundance [62]. Therefore, the *Gemmatimonadetes* composite genome recovered from this switchgrass-adapted community represents an unexplored branch of this phylum, as it is enriched under thermophilic conditions in liquid media. Inspection of the *Gemmatimonadetes* genome revealed that it has multiple glycoside hydrolases responsible for cellulose and hemicellulose hydrolysis as well as a number of multicopper oxidase genes predicted to be laccases, possibly involved in lignin oxidation. The predicted role for *Gemmatimonadetes* in biomass deconstruction was further supported by subsequent proteomic analysis (see below).

In addition to *Gemmatimonadetes*, the *Paenibacillus* and *Rhodothermus* populations were predicted by the metagenome to be involved in biomass deconstruction. The reconstructed *Paenibacillus* genome has a large number of genes for biomass deconstruction, including a complete set of genes for cellulose hydrolysis (cellobiohydrolase, endoglucanase, β-glucosidase). The presence of a broad complement of glycoside hydrolases in the *Paenibacillus* population is consistent with genomes of related Gram-positive *Firmicutes* such as *Paenibacillus* sp. strain JDR-2 (http://www.cazy.org/b991.html). The reconstructed genome for the *Rhodothermus* population has a very similar gene content and high amino acid identity (96%) to the genome of *Rhodothermus marinus*, isolated from a submarine alkaline hot spring in Iceland [63]. This thermophile has been shown to secrete endoglucanase, xylanase and alpha-L-arabinofuranosidase enzymes when grown in the presence of biomass substrates [64]–[66]. A remarkable aspect of the comparison between the reconstructed *Rhodothermus* genome from the switchgrass-adapted consortium and the *R. marinus* genome is that they are so similar despite being recovered from highly divergent environments.

While metagenomic sequences outlined the broad metabolic capabilities of the abundant populations present in the switchgrass adapted community, proteomic data allowed us to focus on the pathways that are actually expressed, and refine the assignment of roles for community members in biomass deconstruction. Proteins for the deconstruction of starch (GH13, GH31) were abundant and had a broad distribution in the proteome. GH13 proteins were detected from *Paenibacillus*, *Rhodothermus*, *Sphaerobacter* and *Thermus*. This observation is consistent with the presence of water-insoluble starch in the switchgrass, which had been extracted with hot water to remove soluble components [8]. The amorphous starch present, a non-structural component of switchgrass, would be readily accessible to enzymes in comparison to cellulose. The majority of the other glycoside hydrolases detected by proteomics were involved in the deconstruction of hemicellulose. A GH74 family putative xyloglucanase from *Sphaerobacter* is the most abundant overrepresented glycoside hydrolase in the supernatant, followed by an alpha-L-arabinofuranosidase (GH51) from *Paenibacillus*, and xylanases (GH10) from *Paenibacillus* and *Hyphomicrobium*. This observation is consistent with the high levels of xylanase activity observed in the supernatant of the switchgrass and supports a model in which xylan is the primary polysaccharide that is hydrolyzed by the switchgrass adapted community during growth. In contrast, only one cellulase, an endoglucanase (GH5) from *Paenibacillus*, was abundant and overrepresented in the supernatant, which mirrored the relatively low level of endoglucanase activity recovered from the switchgrass-adapted community. Comparison of the supernatant proteome of the switchgrass-adapted community with the proteome of the same consortium that had been subsequently perturbed by cultivation with microcrystalline cellulose [9] demonstrated that the complement of glycoside hydrolases was different for the cellulose community. In particular, in the supernatant from the cellulose-perturbed community, a cellobiohydrolase (GH48), two endoglucanases (GH5 and GH9), a xylanase (GH10) and an alpha-L-arabinofuranosidase were detected from *Paenibacillus* that were not detected in the switchgrass-adapted cultures. These results indicate that bacteria have a differential response to the composition of biomass substrates, a phenomenon which has also been observed in quantitative proteomic analysis of *Clostridium thermocellum* and *Caldicellulosiruptor obsidiansis* [67], [68].

A number of unexpected proteins were detected in the supernatant proteome that hint at unexplored steps in lignocellulose deconstruction by bacteria. Sugar isomerases were overrepresented in the supernatant proteome and detected from multiple community members, despite their documented roles in cytoplasmic sugar catabolism. These detections may result from cell lysis during the two week cultivations; however, extracellular xylose isomerases have been found in *Streptomyces* sp. (strain NCL 82-5-1) [69] and a thermophilic *Bacillus* sp. (NCIM 59) [70], and have also been observed at high relative levels in *C. obsidiansis* grown on acid-treated switchgrass. Therefore the sugar isomerases may have an extracellular role in transforming monosaccharides produced from hemicellulose hydrolysis, potentially as a means of relieving product inhibition on the primary hemicellulases, or as a way to circumvent competition for these monosaccharides from other members of the microbial community. A second set of unexpected proteins in the supernatant proteome were superoxide dismutase and Mn-catalase, two proteins that detoxify oxygen radicals. Though these proteins are often associated with intracellular processes, recent iTRAQ proteomic analyses of the supernatant of lignin-grown *Thermobifida fusca* demonstrate that both of these proteins were present in the supernatant [71]. The presence of these proteins suggests that oxygen radicals are produced by the microbes in the extracellular medium, and these radicals may be involved in lignin deconstruction. The detection of only one possible lignin deconstructing enzyme in the supernatant proteome, a laccase from *Gemmatimonadetes,* provides further evidence for our current lack of understanding of bacterial lignin deconstruction.

In conclusion, we have used proteogenomics to propose roles for individual community members in the deconstruction of switchgrass by a thermophilic bacterial consortium. From the metagenomics analysis, we were able to reconstruct multiple nearly complete draft genomes, including the genome of an uncultivated lineage in the *Gemmatimonadetes*. Proteomic analysis indicated the central role played by *Paenibacillus* in biomass deconstruction and confirmed that hemicellulose hydrolysis was a primary activity of the switchgrass-adapted community.

## Supporting Information

File S1 **Scaffold name, IMG Object ID, read coverage, GC%, length, and phylogenetic bin assignment for all the scaffolds in the assembled metagenome.**
(XLSX)

File S2 **Comparison of average read coverage of the metagenomic clusters versus 16S rRNA composition of the consortium, with and without correcting for average 16S rRNA copy number in sequenced reference genomes.**
(XLSX)

File S3 **Completeness of the genome coverage within the metagenomic clusters, estimated based on presence of 54 single-copy or almost single-copy phylogenetic COGs.**
(XLSX)

File S4 **Lignocellulolytic enzymes identified based on dbCAN** **[25]** **, blastp against a local copy of the CAZy** **[26]** **and FOLy** **[27]** **databases (E<1e^−20^), and bacterial laccases** **[28]**, **[29]** **.** Also includes a table of CAZy enzymes found per phylogenetic bin, and a comparison of glycoside hydrolase content in termite hindgut [72], cow rumen [12], a switchgrass adapted compost community [7], leafcutter ant garden [73], and anaerobic microbial community decomposing poplar wood chips [74], and thermophilic cellulose-degrading sludge [75].
(XLSX)

File S5 **Peptides and proteins detected in all three fractions of the proteome, normalized protein abundances, corresponding gene annotations, proteins overrepresented in the supernatant, and comparison of sugar isomerase abundance across the three fractions.**
(XLSX)
